# Adipose tissue fatty acid chain length and mono-unsaturation increases with obesity and insulin resistance

**DOI:** 10.1038/srep18366

**Published:** 2015-12-17

**Authors:** Chong Yew Tan, Samuel Virtue, Steven Murfitt, Lee D. Robert, Yi Hui Phua, Martin Dale, Julian L. Griffin, Francisco Tinahones, Philipp E. Scherer, Antonio Vidal-Puig

**Affiliations:** 1 University of Cambridge Metabolic Research Laboratories, Institute of Metabolic Science, MDU MRC. Addenbrooke’s Hospital, Cambridge, CB2 0QQ; 2 University of Cambridge Department of Biochemistry, 80 Tennis Court Road, Cambridge, CB2 1GA; 3 UGC Endocrinologia y Nutrición (IBIMA), Hospital Virgen de la Victoria. CIBER of Physiopathology, Obesity and Nutrition (CIBEROBN) Málaga, Spain; 4 Touchstone Diabetes Center, Department of Internal Medicine, University of Texas Southwestern Medical Center, Dallas, Texas, USA; 5 Wellcome Trust Sanger Institute, Hinxton, Uk; 6 Medical Research Council – Human Nutrition Research, Elsie Widdowson Laboratory, 120 Fulbourn Road, Cambridge, CB1 9NL, Uk

## Abstract

The non-essential fatty acids, C18:1n9, C16:0, C16:1n7, C18:0 and C18:1n7 account for over 75% of fatty acids in white adipose (WAT) triacylglycerol (TAG). The relative composition of these fatty acids (FA) is influenced by the desaturases, SCD1-4 and the elongase, ELOVL6. In knock-out models, loss of SCD1 or ELOVL6 results in reduced Δ9 desaturated and reduced 18-carbon non-essential FA respectively. Both Elovl6 KO and SCD1 KO mice exhibit improved insulin sensitivity. Here we describe the relationship between WAT TAG composition in obese mouse models and obese humans stratified for insulin resistance. In mouse models with increasing obesity and insulin resistance, there was an increase in scWAT Δ9 desaturated FAs (SCD ratio) and FAs with 18-carbons (Elovl6 ratio) in mice. Data from mouse models discordant for obesity and insulin resistance (AKT2 KO, Adiponectin aP2-transgenic), suggested that scWAT TAG Elovl6 ratio was associated with insulin sensitivity, whereas SCD1 ratio was associated with fat mass. In humans, a greater SCD1 and Elovl6 ratio was found in metabolically more harmful visceral adipose tissue when compared to subcutaneous adipose tissue.

Adipocytes are characterized by the presence of a large triglyceride (TAG) rich lipid droplet bounded by a monolayer of phospholipids^1^. As a biological fuel source, TAG has twice the caloric density as an equivalent mass of protein or carbohydrate. Obesity is a result of chronic positive energy balance and is characterized by the accumulation of TAG rich adipocytes. TAG is a neutral lipid consisting of a glycerol moiety esterified with three fatty acids. In humans, the most abundant fatty acids esterified to TAG, in order of decreasing amount are, oleate (C18:1n9), palmitate (C16:0) and linoleate (C18:2n6). These three FFAs make up around 85% of all TAG. The next most abundant FFAs are palmitoleate (C16:1n7), stearate (C18:0) and vacceneate (C18:1n7), which in turn account for a further 8% of FFAs^2^,^3^,^4^. The five non-essential fatty acids; oleate, palmitate, palmitoleate, stearate and vacceneate, in combination, account for over 75% of all fatty acids found in white adipose tissue^2^,^3^,^4^. Notably, both humans and mice express the three enzymes necessary for *de novo* synthesis of these five non-essential fatty acids; fatty acid synthase (FAS) which performs the *de novo* lipogenesis of C16:0 from acetyl-CoA, Elongation of very long chain fatty acid 6 (ELOVL6), which elongates C16 fatty acids to C18 fatty acids and stereoyl CoA desaturases which desaturate C16, C18 and to a lesser extent, C14 fatty acids^5^,^6^.

In 2002 and 2007, global knock-out mice deficient in the enzymes SCD1^7^ and ELOVL6^8^ were described. These models demonstrate that a loss of SCD1 or ELOVL6 results in the expected changes in tissue fatty acid composition; SCD1 and ELOVL6 knock-out mice were found to have proportionally less Δ9 desaturated and less 18-carbon fatty acids in their tissues respectively. In both these models, the alteration in tissue fatty acids were associated with improved insulin sensitivity and resistance to diet-induced obesity. Some caveats were raised about the fact that SCD1 KO mice were hypermetabolic due to a skin barrier defect. Hypermetabolism has been shown to protect animals from the development of obesity^9^. However, more recently, a liver-specific knock out of SCD1 also demonstrated an improved metabolic profile^10^, suggesting that in both cases, loss of the capacity to generate C18 and Δ9 desaturated non-essential fatty acids was metabolically protective.

In some respects, the finding that mice lacking SCD1 and Elovl6 were protected from metabolic complications was surprising. The principal product of SCD and Elovl6 is oleate, which comprises nearly 45% of the FFA in human adipose tissue. In contrast, there is a considerable body of literature suggesting that diets containing proportionally higher amounts of oleate, as opposed to saturated fats such as palmitate, are protective in terms of insulin sensitivity^11^,^12^. While there have been some studies investigating the relationship between serum FFA composition and associations to the Metabolic Syndrome (MetS), only a small number of studies have investigated changes in SCD ratio in adipose tissue fatty acids^13^,^14^,^15^ and to our knowledge, none have investigated changes in elongation.

In this paper, we ask the question whether progressive weight gain and the development of obesity and insulin resistance is associated with changes in fatty acid composition in white adipose tissue (WAT) triacylglycerols. Our results show that in mouse models of high fat diet and genetic obesity, weight gain and insulin resistance are associated with an increasing proportion of Δ9 desaturated and 18-carbon fatty acids in WAT. We further demonstrate, using obese but insulin-sensitive leptin-deficient mice over-expressing adiponectin (AdTG)^16^ and AKT2 KO mice^17^, that the changes in WAT fatty acid chain length are associated with the degree of insulin resistance whereas changes in desaturation may be more dependent on the accumulation of fat mass. Finally, we show that human visceral fat, which is associated with negative metabolic outcomes, has longer more unsaturated fatty acids.

## Results

### Definition of Elovl6 and SCD activity ratios

**Figure 1:**
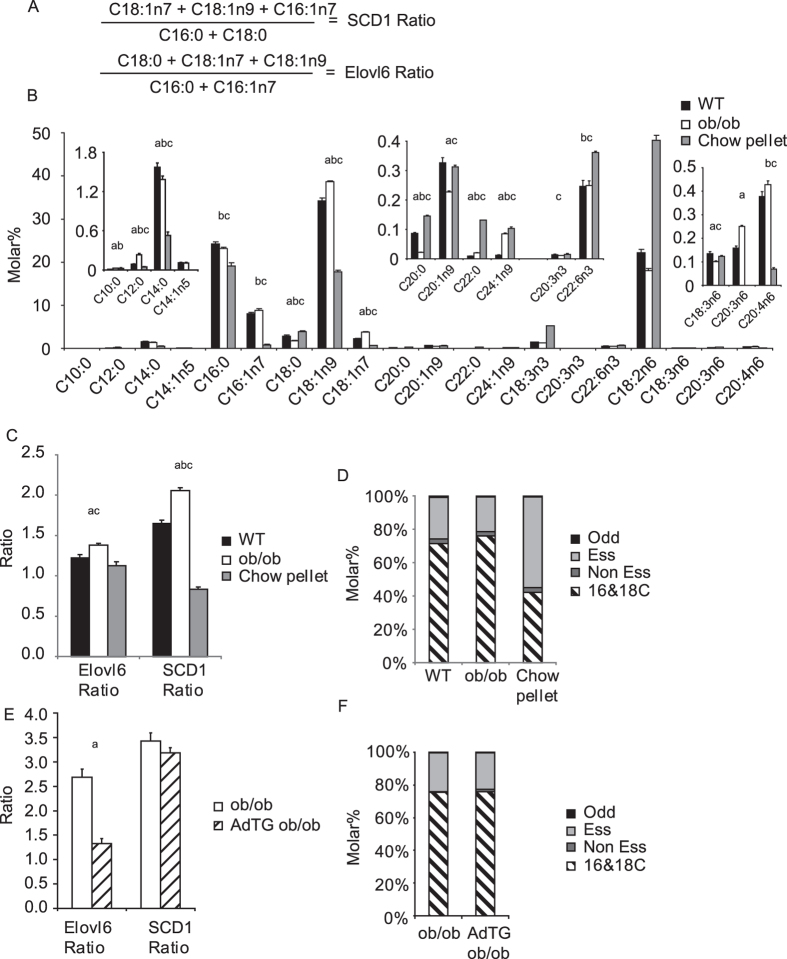
(**A**) Equations for Elovl6 and SCD activity ratios. (**B**) FAME composition in molar percentage in order of increasing chain length and desaturation and classified as non-essential or essential fatty acids. n3 and n6 denotes omega-3 and omega-6 fatty acids respectively. ScWAT FAME composition from 4 month old male C57BL/6 mice (n = 7), ob/ob mice (n = 8) and chow pellets. (**C**) Calculated Elovl6 and SCD ratio from data shown in (**B**). (**D**) Composition of scWAT classified by fatty acid type from data shown in (**B**). (**E**) Elovl6 and SCD ratios for ob/ob and Ad-TG ob/ob scWAT samples (n = 5 per group). (**F**) Composition of ob/ob and Ad-TG ob/ob scWAT lipids classified by fatty acid type. All error bars are 1 S.E.M. Abbreviations: odd chain (Odd) Essential fatty acids (Ess) Non-essential fatty acids excluding C16 and C18 species (Non Ess) and C16 and C18 non-essential fatty acids (16&18C). (**A**) P < 0.05 between genotypes, (**B**) P < 0.05 between chow fed and chow diet, (**C**) P < 0.05 between ob/ob and chow diet.

The two major substrates of SCD are C18:0 and C16:0, while its major products are C18:1n9 and C16:1n7. The two major substrates for Elovl6 are C16:0 and C16:1n7, while its major products are C18:0 (which is subsequently desaturated to C18:1n9) and C18:1n7. We defined the ratio for either SCD or Elovl6 action as the sum of all of its products divided by the sum of all its substrates (Fig. 1A).

### Assessment of DNL rates

To assess DNL in our samples we considered both the traditional C16:0/C18:1n6 ratio and a ratio of the total amount of non-essential fatty acids to essential fatty acids as a second surrogate readout for DNL, which would exclude the confounding effects of palmitate elongation and desaturation.

### Adipose tissue fatty acids chain length and saturation increases in genetic models of obesity

Figure 1B shows the fatty acid composition of subcutaneous white adipose tissue (scWAT) from 4 month old ob/ob mice and lean wild-type mice as well as the fatty acid composition of chow diet pellets. The chow diet had a greater proportion of the PUFA C18:2n6 than of any other single fatty acid, whilst the molar percentage of C18:2n6 was lower than oleate and palmitate in the scWAT of both mouse models (Fig. 1B). We quantified the ratio between 18:2n6 and C16:0 and detected a significantly higher ratio in the ob/ob compared to controls. Our second index of DNL, all non-essential fatty acids divided by all essential fatty acids was also significantly increased in ob/ob mice compared to controls (Supplemental Figure 1B). Of note the 18:2n6/C16:0 and the NE/E indices gave different values (17% increase and 29% increase respectively).

We next analysed the SCD and Elovl6 ratios of the chow diet and the scWAT of the wild type and ob/ob mouse models. ScWAT from ob/ob mice had an increase in both Elovl6 and SCD ratio (Fig. 1C) compared to controls. In line with the reported increase in DNL in ob/ob mice^18^,^19^ and the increase in DNL indices we observed, there was a small but significant increase in the proportion of non-essential fatty acids in ob/ob mice compared to wild type (Fig. 1D).

### Severely obese ob/ob mice over expressing adiponectin (AdTG) have a comparable Elovl6 ratio to lean wild-type mice

The results from the ob/ob mouse raised the question as to whether the increases in Elovl6 ratio and SCD ratio were driven simply by fat mass accretion and/or by insulin resistance. In 2006, Kim *et al.* demonstrated that transgenic mice over expressing adiponectin (AdTG) on an ob/ob background had improved metabolic health in terms of glucose homeostasis when compared to ob/ob littermates. However, AdTG mice were also found to be significantly more obese than ob/ob littermates^16^. Mice over expressing adiponectin had lower Elovl6 ratios compared to ob/ob littermates, but comparable SCD ratios (Fig. 1E). Interestingly, the reversal of Elovl6 ratio towards wild-type levels in AdTG ob/ob occurred via a reduction in scWAT oleate and an accumulation of palmitoleate (C16:1n7) (supplemental Figure 1A). These changes occurred without significant alterations in levels of fatty acid classes (Fig. 1F), or alterations in indexes of DNL (supplemental Figure 1C).

### AKT2 KO mice had longer fatty acids without changes in saturation index

**Figure 2:**
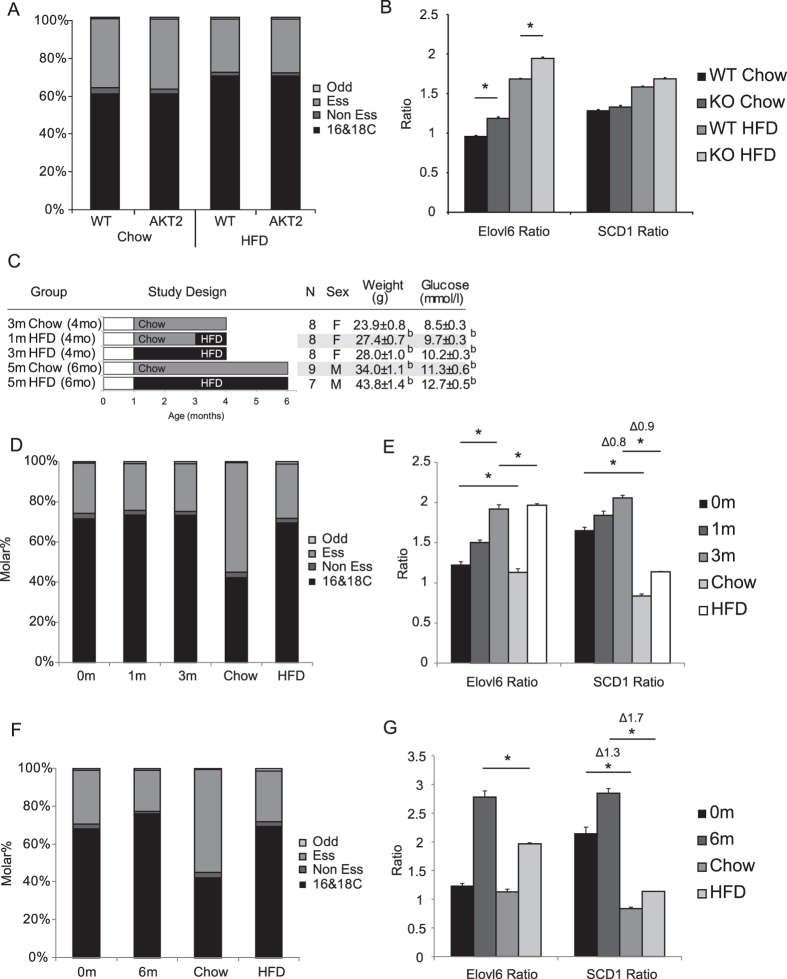
(**A**) Composition of scWAT lipids, classified by fatty acid type for WT or AKT2 KO mice on chow or HFD for 4 months post weaning (**B**) Calculated Elovl6 and SCD ratios for WT or AKT2 KO mice on chow or HFD 4 months post weaning. **(C**) Description of mouse dietary regimes, body weights and blood glucoses. (**D**) Composition of scWAT lipids, classified by fatty acid type for mice on chow and HFD for 0, 1 or 3 months compared with chow and high-fat diet pellets. (**E**) Calculated Elovl6 and SCD ratios for mice on HFD for 0, 1 or 3 months. (**F**) Composition of scWAT lipids classified by fatty acid type for mice on chow or HFD for 0 or 5 months compared with chow and high-fat diet pellets. (**G**) Calculated Elovl6 and SCD ratios for mice on HFD for 0 or 5 months. Error bars indicate 1 S.E.M. (**A**) p(**A**) < 0.05, compared to 0m-HFD fed mice based on t-test (**B**) p < 0.05, compared to 5m-Chow mice based on t-test. *p < 0.05 based on t-test.

The results from the AdTG mice suggested that elongation may be more related to insulin sensitivity and desaturation to adiposity. To investigate this hypothesis further we next considered the AKT2 KO mouse model, a lean mouse that has congenic insulin resistance^17^. AKT2 KO mice did not show any significant changes in fatty acid classes (Fig. 2A) but did exhibit increased levels of oleate and stearate and decreased levels of palmitate and palmitoleate (supplemental Figure 4A). As a result, AKT2 KO mouse on either a chow or a high fat diet had an increased Elovl6 ratio but no change in SCD ratio (Fig. 2B). In terms of DNL indexes the AKT2 KO mice had significantly lower C18:2/C16:0 ratios than wild-types when on a HFD, whereas the NE/E index was similar compared to controls (Supplemental Figure 4B). It was notable that high-fat feeding increased the Elovl6 and SCD ratios as well as both indexes of DNL for both the WT and the AKT2 KO mice substantially over their relative chow groups, however these results did not consider the potential impact of dietary composition on adipose tissue.

### Adipose tissue fatty acids chain length and saturation increases following high fat diet induced obesity

We next sought to determine what the impact of high-fat feeding on Elovl6 and SCD ratios in adipose tissue. Figure 2C shows all the dietary regimes used as well as the weights and blood glucoses of the mice. The analysis of diet-induced obesity models was subject to a strong potential confounding factor, which was the lipid composition of the diets. The lipid in the standard chow diet we used was 100% soy bean oil, whereas the high-fat diet used was 90% lard and 10% soy bean oil. This difference in lipid composition is most clearly shown by the much greater proportion of essential fatty acids (predominantly 18:2n6) in the chow diet compared to the high-fat diet (Fig. 2D and supplemental Figure 2A). To account for the effects of different diets, we analyzed the lipid composition of the diets alongside the lipid composition of the scWAT of the different models.

We first considered relatively short term dietary interventions of 1 month and 3 months HFD feeding (Fig. 2E). In these models, the Elovl6 ratio of chow-fed (0m-HFD) or 3 month HFD fed (3m-HFD) mice was comparable to their respective diets. The 1 month HFD fed (1m-HFD) animals had an intermediate Elovl6 ratio between 0m-HFD and 3m-HFD animals. The SCD ratio showed a subtly different pattern. In both 0m-HFD and 3m-HFD fed mice, the scWAT fatty acid composition was more desaturated than their respective diets. However, the relative increase in desaturation between adipose tissue and the diets consumed remained similar; chow fed mice had an adipose tissue lipid composition 0.8 SCD units larger than their diet, whilst high-fat fed mice had a SCD ratio 0.9 SCD units larger than their diet (Fig. 2E).

To determine the effects of a more severe diet-induced obesity challenge we next investigated 6 month old mice fed either chow or high-fat diet for 5 months from weaning (Fig. 2F,G). In this case, for both SCD and Elovl6 ratios, the ratio found in the scWAT of HFD fed mice was much greater than the diet; the difference between HFD-scWAT and the composition of the HFD was greater than the difference between chow-scWAT and chow diet (as in Fig. 2E). In terms of the specific fatty acids, the increase in fatty acid chain length and desaturation ratios was principally driven by an increase in the proportion of oleate and a decrease in the proportion of palmitate (supplemental Figure 2A).

In terms of DNL rates we saw a relatively consistent pattern, with both the C16:0/C18:2 ratios and the NE/E ratios for chow fed mice consistently exceeding that of the diet whereas for the high-fat fed mice the ratios were largely similar to diet (Supplemental Figure 2B,C). The only discrepancy was that the adipose NE/E ratio slightly exceeded the dietary NE/E ratio after 1 and 3 months of high fat feeding (Supplemental Figure 2B,C).

### Visceral WAT contained longer more monounsaturated fatty acids than scWAT in morbidly obese humans

In an attempt to translate these findings to humans, we set out to investigate the relationship between insulin sensitivity and white adipose tissue Elovl6 and SCD ratios. Morbidly obese non-diabetic subjects matched for body mass index (BMI), age and gender, but discordant for insulin sensitivity as assessed by HOMA-IR were studied (See table 1 for subject characteristics).

**Figure 3:**
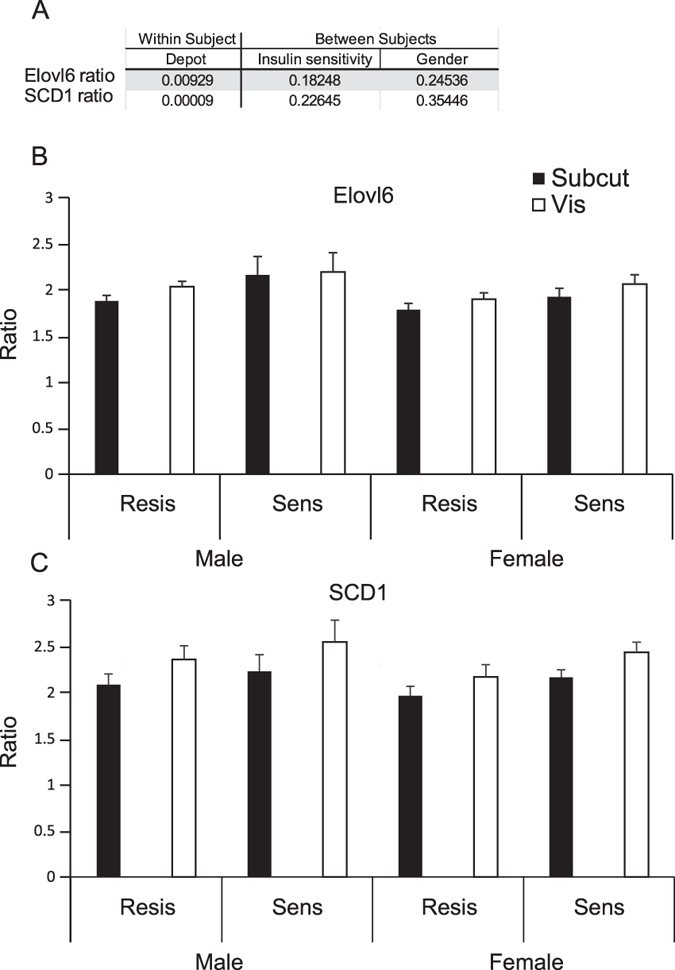
(**A**) Table showing p-values from mixed model ANOVA using WAT depot as within subject factor with gender and insulin sensitivity as between subject factors. (**B**) Elovl6 ratios for subjects stratified by gender and insulin resistance status for visceral and subcutaneous adipose tissue. (**C**) SCD ratios for subjects stratified by gender and insulin resistance status for visceral and subcutaneous adipose tissue. Values are mean +/− 1 S.E.M.

There were 8 sub-groups of subjects based on gender (Male or Female), HOMA-IR (Sensitive or Resistant), and depot (scWAT or vWAT). For ScWAT and vWAT fatty acid composition see supplemental Figure 3A. We analyzed Elovl6 (Fig. 3B) and SCD (Fig. 3C) ratios for effects of gender, insulin sensitivity and depot and found that only depot was significant (Fig. 3A). Both Elovl6 and SCD ratios were persistently greater in vWAT compared to scWAT regardless of gender or insulin sensitivity.

In terms of DNL indices, vWAT showed a greater NE/E ratio than scWAT (supplemental Figure 3B).

### Elovl6 has a greater specificity for palmitate than palmitoleate

In our data set we possessed information regarding the location of the double bond in C18:1. It was therefore possible to distinguish fatty acids derived from elongation of palmitate (C18:0 and C18:1n9) and those elongated from palmitoleate (C18:1n7). We generated a further ratio for palmitate elongation (C18:0+C18:1n9)/C16:0 and palmitoleate elongation C18:1n7/C16:1n7. By dividing the palmitate elongation equation by the palmitoleate elongation equation we were able to determine that palmitate was the preferential substrate for Elovl6, with Elovl6 appearing to have over 4 times the preference for palmitate than palmitoleate in both mice and humans (supplemental Figure 5A). It was notable that in ob/ob mice Elovl6 had a lower apparent specificity for palmitate than WT mice (Supplemental Figure 5A) whereas in AdTG-ob/ob mice Elovl6 had a much greater apparent specificity for palmitate than ob/ob controls (Supplemental Figure 5C).

### Fatty acid elongation and desaturation occur predominantly in liver in genetic models of obesity

**Figure 4:**
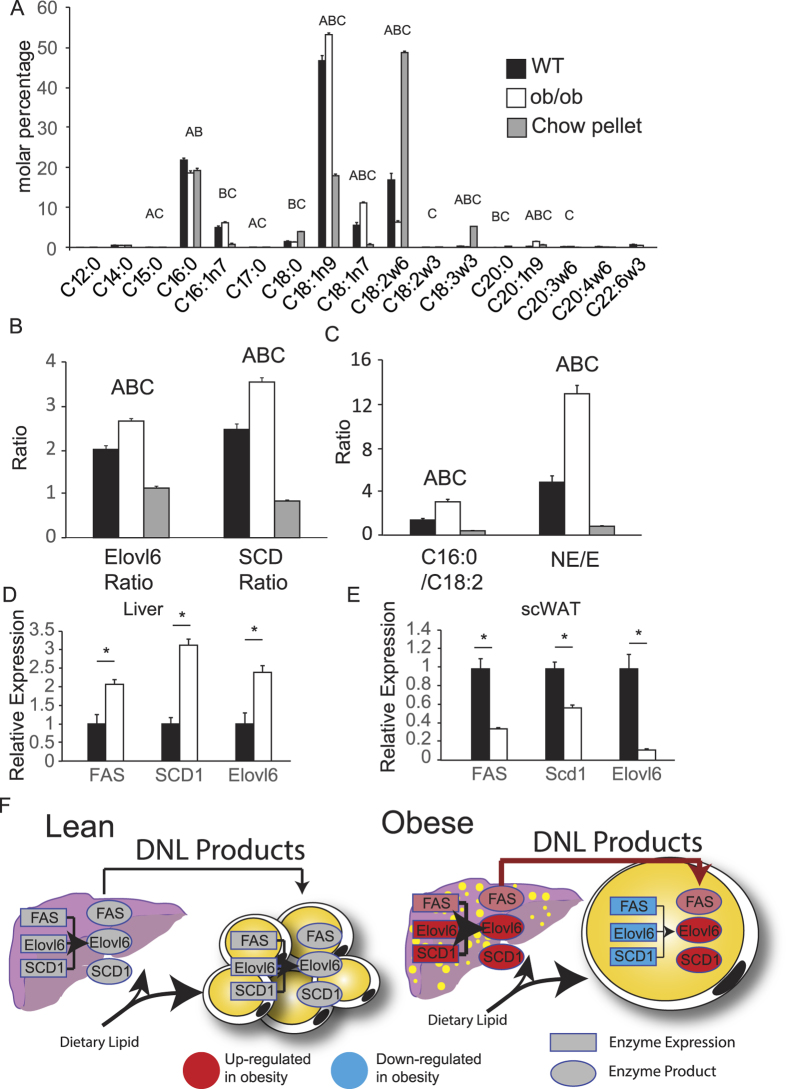
(**A**) Liver triglyceride and chow diet fatty acid profiles (**B**) Liver TG and chow diet Elovl6 and SCD1 ratios (**C**) Liver TG and chow diet indices of DNL (**D**) Gene expression from liver (**E**) Gene expression from scWAT. (**F**) Summary figure highlighting changes in enzymes and their products in liver and adipose tissue in lean and obese states. Boxes represent enzyme expression levels, ovals represent enzyme products. Red boxes represent upregulation, blue represent down regulation and grey represent baseline levels of expression and concentration. (**A**) P < 0.05 between genotypes, (**B**) P < 0.05 between chow fed and chow diet, (**C**) P < 0.05 between ob/ob and chow diet. *P < 0.05. Values are mean +/−1 S.E.M.

To try to determine whether fatty acid elongation was occurring in adipose tissue itself or liver we profiled the triglyceride pool of WT and ob/ob livers (Fig. 4A). We determined that TAG in the livers of WT or ob/ob mice had Elovl6 and SCD ratios that were nearly twice the level of their respective adipose tissue ratios (Fig. 4B). Furthermore, indices of DNL indicated that liver TAG appeared to derive from DNL to a proportionately much greater extent in liver than in adipose tissue (Fig. 4C). It was notable that in the ob/ob mouse livers, when compared to the wild-type, there were greater inductions in Elovl6, SCD1 and DNL ratios than those seen for adipose tissue. We profiled mRNA from liver and scWAT and demonstrated that, when compared to the wild-types, ob/ob miced had increased Elovl6, FAS and SCD1 in livers (Fig. 4D) but these genes were decreased in scWAT (Fig. 4E).

## Discussion

In this study we describe the association between adipose tissue lipid composition and the development of obesity and metabolic dysfunction in mouse models of obesity and obese human subjects. We found that increasing obesity/metabolic dysfunction was associated with an increase in the proportion of C18 and Δ9 desaturated lipids within adipose tissue. The two most common models for the investigation of obesity in mice are the diet-induced obesity model and the ob/ob model of genetic obesity. We initially studied ob/ob mice to avoid any confounding effects of changes in diet on the lipid profile of the adipose tissue. Leptin-deficient ob/ob mice exhibited a fatty acid profile in adipose tissue that was both longer and more desaturated than the wild-type controls. The increased FA SCD and elongation ratios in ob/ob mice could have been related to fat mass accretion or insulin resistance. To uncouple fat mass gain from metabolic complications we next studied the AdTG-ob/ob mouse model, which over-expresses the globular domain of adiponectin under an aP2 promoter. AdTG ob/ob-mice are more obese than ob/ob controls, but are much more insulin sensitive. Strikingly, AdTG mice had similar SCD ratios to ob/ob mice, but dramatically reduced Elovl6 ratios. In contrast, AKT2 KO mice, which are insulin resistant without being obese, had an increased Elovl6 ratio relative to wild-type controls, but no difference in SCD ratio. These results demonstrated two important points; first SCD and Elovl6 ratios are not necessarily co-regulated. This was in contrast to the observation that Elovl6 and SCD ratios increased in tandem in both the ob/ob and dietary models of obesity. Second, the fact the Elovl6 ratio was lower when comparing the AdTG mouse to the ob/ob and increased in AKT2 KO mice compared to wild-type controls suggested that the Elovl6 ratio may primarily relate to insulin sensitivity rather than fat mass, however evidence from more than just two mouse models will be needed to substantiate this finding. Mechanistically, how Elovl6 and SCD1 may be divergently regulated remains to be determined, however at least one study has reported that loss of AKT2 specifically leads to upregulation of Elovl6 and not SCD1 in liver^20^, suggesting that there may be divergent transcriptional programs for these enzymes.

In addition to assessing Elovl6 and SCD ratios we also looked at two indices of DNL. The ratio C16:0/C18:2 has been used previously, however given that we had described increases in both Elovl6 and SCD products we generated an alternative measure using the sum of the molar percentages of the non-essential fatty acids and dividing it by the sum of the essential fatty acids. It was notable that these two measures gave slightly different results, with the NE/E ratio suggesting a greater increase in DNL in the ob/ob mouse compared to the C16:0/18:2n6 ratio. This observation was consistent with the fact both the SCD and Elovl6 ratios were increased in the ob/ob mouse, which would reduce the amount of C16:0 and therefore the C16:0/C18:2 ratio. Importantly, the NE/E ratios for both the AKT2 KO mouse and the AdTG mouse were similar to their respective controls, suggesting that changes in Elovl6 ratio occurring in these mice were doing so independently of apparent changes in DNL. One final set of ratios we considered were the ratios for the action of Elovl6 on palmitate or palmitoleate. Assessing these ratios showed that Elovl6 preferentially elongated palmitate rather than palmitoleate. It was notable that when Elovl6 activity was high (in the ob/ob vs the WT) the specificity of Elovl6 for palmitate over palmitoleate was diminished, whereas when Elovl6 activity appeared to be limited (Ad-TG ob/ob vs ob/ob), the specificity of Elovl6 for palmitate was greatly increased. These data suggest that palmitate may act as a competitor for palmitoleate, and when Elovl6 activity is low, palmitate may inhibit the elongation of palmitoleate (Supplemental Figure 5 for a diagramatic representation). Importantly, the fact elongation of palmitoleate varies inversely with Elovl6 activity makes the C18:1n7/C16:1n7 ratio a poor readout of Elovl6 activity.

We next investigated the effects of high fat feeding. While previous studies have investigated the composition of fatty acids in adipose tissue from chow controls and high-fat fed mice, they have failed to control for the effect of dietary fatty acid composition. This important confounding factor makes conclusions about the impact of high-fat feeding on Elovl6 and SCD activity difficult to interpret. In our study we found that the differential in the Elovl6 and SCD ratios of scWAT, when compared to diet, only exceed those of chow fed mice after 5 months of HFD feeding. Combined with the data from the ob/ob model, this result suggests that a prolonged and severe metabolic challenge was required for fatty acids in adipose tissue to demonstrate a level of processing that exceeded what occurs in controls.

In terms of DNL ratios chow fed animals had DNL ratios (both C18:2n6 and NE/E) that far exceeded that of their diet. Conversely, in general, high fat diet fed mice had DNL ratios that were similar to that of their diet, consistent with the mice preferentially storing dietary fat and engaging in relatively low levels of DNL.

Finally we considered a cohort of human patients that were classified based on differential HOMA-IR values. In this cohort, the only significant effect that was detected was a difference between the visceral and subcutaneous adipose tissue depots. The fact that visceral adipose tissue has long been considered a more metabolically deleterious adipose tissue depot than subcutaneous adipose tissue and exhibits both longer and more unsaturated non-essential fatty acids was in good accordance with the findings from mouse models of obesity. The lack of association between insulin sensitivity and Elovl6 or SCD ratio may have represented a problem of statistical power. Comparisons between depots were paired and the composition of the depots in terms of their Elovl6 and SCD ratios were highly correlated (r = 0.9 for Elovl6, r = 0.8 for SCD). In contrast, the larger variation in SCD and Elovl6 ratios between subjects (as oppose to between depots within the same subject) may have been influenced by differences in diet or genetic factors; factors which should not have affected intra-subject depot differences. Evidence from a larger cohort of subjects with detailed dietary records will be necessary to determine if our observations from mice regarding SCD1 and Elovl6 ratios in adipose tissue can be extended to humans. In terms of DNL we detected a tendency for the C16:0/C18:2 ratio to be increased and a significant increase in NE/E ratio in vWAT compared to scWAT. The discrepancy between the two measures may be explained by the increased Elovl6 and SCD ratios in vWAT compared to scWAT that would result in reduced palmitate levels and reduced C16:0/C18:2 levels. The exact mechanism underlying differences in Elovl6 and SCD1 ratios between vWAT and scWAT in humans remain to be determined, however at least one report has demonstrated differential release of specific FFAs from scWAT and vWAT, which over time could lead to changes in their composition^21^.

An important question is what do the Elovl6 and SCD ratios actually mean? While these are conceptually a read out of enzymatic activity, they do not necessarily reflect its actions within adipose tissue itself. While one study has associated SCD1 mRNA levels in human adipose tissue to SCD ratios^14^, our data would suggest that elongation and desaturation of fatty acids, at least in mice, may occur predominantly in the liver rather than in adipose tissue. In our study we were able to compare liver lipid and mRNA profiles to scWAT profiles for the wild-type and ob/ob mouse groups. It was notable that even in wild-type mice liver TG was predominantly derived from DNL and then elongated and desaturated to a greater extent than the TG found in scWAT or diet. Adipose tissue appeared to have a profile in terms of Elovl6, SCD1 and DNL indices that fell between the diet and liver, suggesting a mechanism by which the majority of dietary lipid is directed to adipose tissue in the fed state when insulin is high. In addition to lipid from the diet itself, the liver generates fatty acids through DNL and exports these for storage in WAT. The net result is that WAT FFA composition is a hybrid of diet and DNL. In the ob/ob mouse, which is hyperphagic and hyperinsulinaemic, the profiles observed suggest an exaggerated version of this model. Consistent with previous reports ob/ob mice had a lipid profile in liver representative of greater rates of DNL compared to controls^22^,^23^. In addition to increased DNL, ob/ob mice also had a liver lipid profile that exhibited greater amounts of fatty-acid elongation and desaturation compared to wild-types. We have summarized our proposed pathways of changes in heaptic and adipose tissue FAS, Elovl6 and SCD1 in Fig. 4F.

With regards to the location of elongation and desaturation within the mouse, further support was leant to the idea that liver may be the key organ for this process by transcriptional profiles. Despite having longer and more desaturated fatty acids in adipose tissue, ob/ob mice had reduced mRNA expression of Elovl6 and SCD1 compared to controls. Conversely, Elovl6 and SCD1 were upregulated in the livers of ob/ob mice compared to controls.

Overall, our data represent a body of evidence that raises several questions. Firstly, why are diets rich in oleate metabolically protective^11^,^12^,^24^? If oleate accumulation in WAT is a hallmark of obesity and insulin resistance in mice and is more prevalent in metabolically harmful visceral adipose tissue in humans, then the protective effects of oleate-rich diets cannot work by simply increasing the body’s oleate levels. This point is further highlighted by the metabolically healthy phenotypes of SCD1 and Elovl6 KO mice, which both have reduced oleate levels in all tissues studied. One possible explanation is that there is selective substrate partitioning to specific cellular metabolic processes of ‘*de novo*’ synthesised oleate, produced by Elovl6 and SCD action, as opposed to dietary oleate. The concept of selective substrate partitioning for *de novo* synthesized palmitate, produced by FAS, toward the production of phosphatidylcholine has been previously demonstrated^25^,^26^. In line with this concept, oleate rich diets strongly suppress FAS, Elovl6 and SCD1 in liver.

In summary, our data provide a comprehensive overview of the changes in adipose tissue fatty acid composition in murine models of obesity and human metabolic dysfunction. We demonstrate that increased fat mass is associated to greater degree of fatty acid Δ9 desaturation, whereas metabolic dysfunction associates with increased C18 to C16 ratio in adipose tissue.

## Materials and Method

### Animals and diet

All animal breeding and experiments were approved by and conducted in accordance with the UK Home Office scientific procedures act 1986 and the University of Cambridge LRP. Animals were housed in a specific pathogen free facility with 12 hour light and 12 hour dark cycles (Light cycle 0600h to 1800h). All animals were studied at 24 °C and a humidity of 55%. Standard laboratory chow diet (Research Diets D12450B, protein 20%, carbohydrate 70% and fat 10%) and high fat diet (HFD) (Research Diets D12451, protein 20%, carbohydrate 35% and fat 45%) were used in this study. All diets and water were provided *ad libitum*. AKT2 KO mice were generated as previously reported^17^. Wild type and leptin deficient ob/ob mice were on a C57BL/6 background. Sub-cutaneous white adipose tissue from AdTG ob/ob mice were provided by Philip Scherer.

### Human white adipose tissue

All participants gave their informed consent and the study was approved by and conducted in accordance with the ethics and research committee of the Vírgen de la Victoria Clinical University Hospital (Malaga, Spain). Samples were obtained from obese patients undergoing bariatric surgery at the Hospital Clínico Virgen de la Victoria. Exclusion criteria and classification by HOMA-IR were as previously described^19^, briefly insulin resistance was defined as having HOMA-IR above two standard deviations from the mean of a healthy lean controls. Both scWAT and vWAT adipose tissues were obtained at the beginning of the surgical procedure and were stored immediately at -80 °C.

### Fatty acid analysis

Total lipids were extracted from frozen tissue using a modified Folch extraction method. Chloroform:methanol (2:1 v/v) was added to 20 mg of tissues with deuterated tridecanoic acid as an internal standard. Samples were homogenised with ceramic beads (MP biomedicals 6540-434) at 5 Hz for 2 minutes at room temperature. Extracted lipids were dried under nitrogen stream and subsequently saponified and derivatised into fatty acid methyl esters (FAME) using 10% boron trifluoride in methanol. Gas chromatography was performed on a Thermo Finnigan Focus GC coupled to a FID detector or an Agilent 7890B GC connected to a 5977A MSD, both using a Thermo Scientific TR-FAME column (length: 30 m, internal diameter: 0.25 mm, film size: 0.25 μm) with helium as carrier gas (1.5 ml/min). Inlet and FID detector temperature was set at 230 °C and 250 °C respectively, MSD detector temperature was set at 230 °C. Oven programme were as follows: 100 °C 2 min, 25 °C/min to 150 °C, 2.5 °C/min to 162 °C, 162 °C 3.8 min, 4.5 °C/min to 173 C, 173 °C 5 min, 5.0 °C/min to 210 °C, 40 °C/min to 230 °C, 230 °C for 0.5 min. Results were presented and analysed as molar percentage of total FAME.

### Solid phase extraction of liver samples

Folch extracts of liver (~90 mg) were resuspended in chloroform and applied to aminopropyl (NH2) solid-phase extract columns (Agilent, UK). The Flow through was collected along with two subsequent washes with 1 ml chloroform. The flow through contained TAG while phospholipids and FFAs remained on the column. The flow through was dried under nitrogen and derivitised as described above.

### RNA extraction and real time PCR

RNA was extracted using STAT-60 (AMS biotech) according to manufacturer’s procedures. Reverse transcription was performed using Reverse Transcriptase System (Promega) according to manufacturer’s instructions. Real-time PCR was carried out using TaqMan or Sybr Green reagents using an Abi 7900 real-time PCR machine using default thermal cycler conditions. Primers available on request.

All statistics were performed using SPSS 18.0. Statistical significance was set at a p-value <0.05.

## Additional Information

**How to cite this article**: Yew Tan, C. *et al.* Adipose tissue fatty acid chain length and mono-unsaturation increases with obesity and insulin resistance. *Sci. Rep.* **5**, 18366; doi: 10.1038/srep18366 (2015).

## Supplementary Material

Supplementary Figures

## Figures and Tables

**Table 1:** Subject characteristics of morbidly obese individuals undergoing elective bariatric surgery.

	Sensitive		Resistant		p value	
	Male	Female	Male	Female	Group	Gender
N	7	7	7	7	–	–
Age (yrs)	39.9 ± 4.2	48.4 ± 4.8	35.7 ± 3.5	42.2 ± 3.1	0.212	0.061
Weight (Kg)	166.3 ±9.5	140.4 ± 6.7	172.4 ± 9.3	145.6 ± 7.1	0.659	**0.002**
Height (cm)	168.7 ± 2.1	159.4 ± 1.9	174.9 ± 2.3	158.4 ± 3.5	0.386	**0.000**
BMI	58.3 ± 2.5	55.1 ± 1.9	56.3 ± 2.7	57.9 ± 1.0	0.850	0.467
Insulin (pmol/l)	16.8 ± 2.4	14.0 ± 1.2	43.1 ± 4.3	53.5 ± 4.6	**0.000**	0.742
Glucose (mmol/l)	5.3 ± 0.4	5.4 ± 0.2	5.7 ± 0.2	6.4 ± 0.6	**0.021**	0.160
HOMAIR	3.9 ± 0.5	3.3 ± 0.3	10.7 ± 1.1	15.3 ± 2.5	**0.000**	0.423
Cholesterol (mmol/l)	4.9 ± 0.3	5.0 ± 0.4	4.3 ± 0.3	5.6 ± 0.2	0.948	**0.045**
Triglyceride (mmol/l)	1.2 ± 0.2	1.3 ± 0.2	1.5 ± 0.4	1.9 ± 0.4	0.274	0.655
HDL-Chol (mmol/l)	1.0 ± 0.1	1.3 ± 0.1	0.8 ± 0.1	1.4 ± 0.1	0.380	**0.001**
NEFA (mmol/l)	0.6 ± 0.1	0.7 ± 0.1	0.7 ± 0.1	0.6 ± 0.0	0.892	0.800

p values following 2 factor ANOVA with Group (Insulin sensitive or resistant) and gender as independent factors. Insulin sensitivity was defined as having HOMA-IR within 2 standard deviation of a lean healthy population. Values are mean +/−1 S.E.M.
