# Erratum: Adipose tissue fatty acid chain length and mono-unsaturation increases with obesity and insulin resistance

**DOI:** 10.1038/srep23873

**Published:** 2016-03-30

**Authors:** Chong Yew Tan, Samuel Virtue, Steven Murfitt, Lee D. Roberts, Yi Hui Phua, Martin Dale, Julian L. Griffin, Francisco Tinahones, Philipp E. Scherer, Antonio Vidal-Puig

Scientific Reports 5: Article number: 18366;10.1038/srep18366 published online: 12172015; updated: 03302016

The original version of this Article contained a typographical error in the spelling of the author Lee D. Roberts, which was incorrectly given as Lee D. Robert. This has now been corrected in the PDF and HTML versions of the Article.

