# Supplementary material for: Adipose tissue fatty acid chain length and mono-unsaturation increases with obesity and insulin resistance
Source: Sci Rep. 2015 Dec 17;5:18366. doi: 10.1038/srep18366 (PMC4683622; doi:10.1038/srep18366)
Supplement: Supplementary Figures [file srep18366-s1.doc]

**Supplemental information for Adipose tissue fatty acid chain length and mono-unsaturation increases with obesity and insulin resistance**

Chong Yew Tan^1,5^, Samuel Virtue^1,5^, Steven Murfitt^2^, Hui Phua Yi^2^, Martin Dale^1^, Julian Griffin^2^, Francisco Tinahones^3^, Philipp E. Scherer^4^, Antonio Vidal-Puig^1,5^

**Supplemental figure 1** A) Fatty acid methyl ester composition of lipids extracted from whole scWAT of ob/ob and adiponectin-transgenic ob/ob mice (adTG). B) DNL ratios for ob/ob and control mice calculated from Figure 1A C) DNL ratios for ob/ob and adTG mice calculated from Supplemental Figure 1 A) Samples were from 4 month old male C57BL/6 mice fed a chow diet. N=8 per group mice (panel B) or 5 month old male mice (panels A and C) C57BL/6 fed a chow diet. N=5 per group. * P<0.05

**Supplemental figure 2** A) Fatty acid methyl ester composition of lipids extracted from whole scWAT of HFD fed mice. B and C) DNL ratios calculated from A. Samples were from 4 month old male C57BL/6 mice fed HFD for; **0m,** 0 months (n=8), **1m,** 1 month (n=8) and **3m**, 3 months (n=8); 6 months old male C57BL/6 mice fed HFD for **0M,** 0 months (n=9) and **5m,** 5 months (n=7); extracted lipids from chow (n=3) and HFD pellets (n=3). $ P<0.05 ANOVA for 1 and 3 month HFD fed mice and 4 month old chow-fed controls. (All mice 4 months of age) * P<0.05 5 months HFD fed mice vs 6 month old chow fed controls (all mice 6 months of age).

**Supplemental figure 3.** Fatty acid methyl ester composition of lipids extracted from whole scWAT and vWAT of morbidly obese subjects undergoing elective bariatric surgery. FAME composition in molar percentage in order of increasing chain length and desaturation and classified as non-essential or essential fatty acids. n3 and n6 denotes omega-3 and omega-6 fatty acids respectively. No significant effect of insulin sensitivity (Resistant versus Sensitive) or gender (Male versus Female) was found with two factors ANOVA. A) FAME composition of scWAT. B) FAME composition of vWAT C) DNL ratios calculated from A and B, D) Significance values from repeated measures ANOVA for DNL indexes.

**Supplemental figure 4** A) Fatty acid methyl ester composition of lipids extracted from whole scWAT of WT or AKT2 KO mice. B) DNL ratios calculated from A) for Samples were from 5 month old male C57BL/6 fed a chow or HFD from weaning. N=8 per group. A, P<0.05 for diet, B P<0.05 for genotype C P<0.05 Diet*Genotype interaction from a two-way ANOVA. * P<0.05 for t-test for DNL ratios (effects were not compared between diets due to different C16:0 and 18:2n6 compositions in the diet).

**Supplemental figure 5** A) Palmitate elongation ratio/palmitoleate elongation ratio ((C18:0+C18:1n9):C16:0):(C18:1n7:C16:1n7) for wt and ob/ob mice as well as human patient groups. B) Palmitate elongation ratio/palmitoleate elongation ratio for AdTG-ob/ob and ob/ob control mice. C) Diagram describing changes in palmitate vs palmitoleate elongation in different mouse models. Strength of black arrows = level of enzymatic activity. Coloured arrows represent significant changes in FFA levels (blue down, red up). *P<0.05.


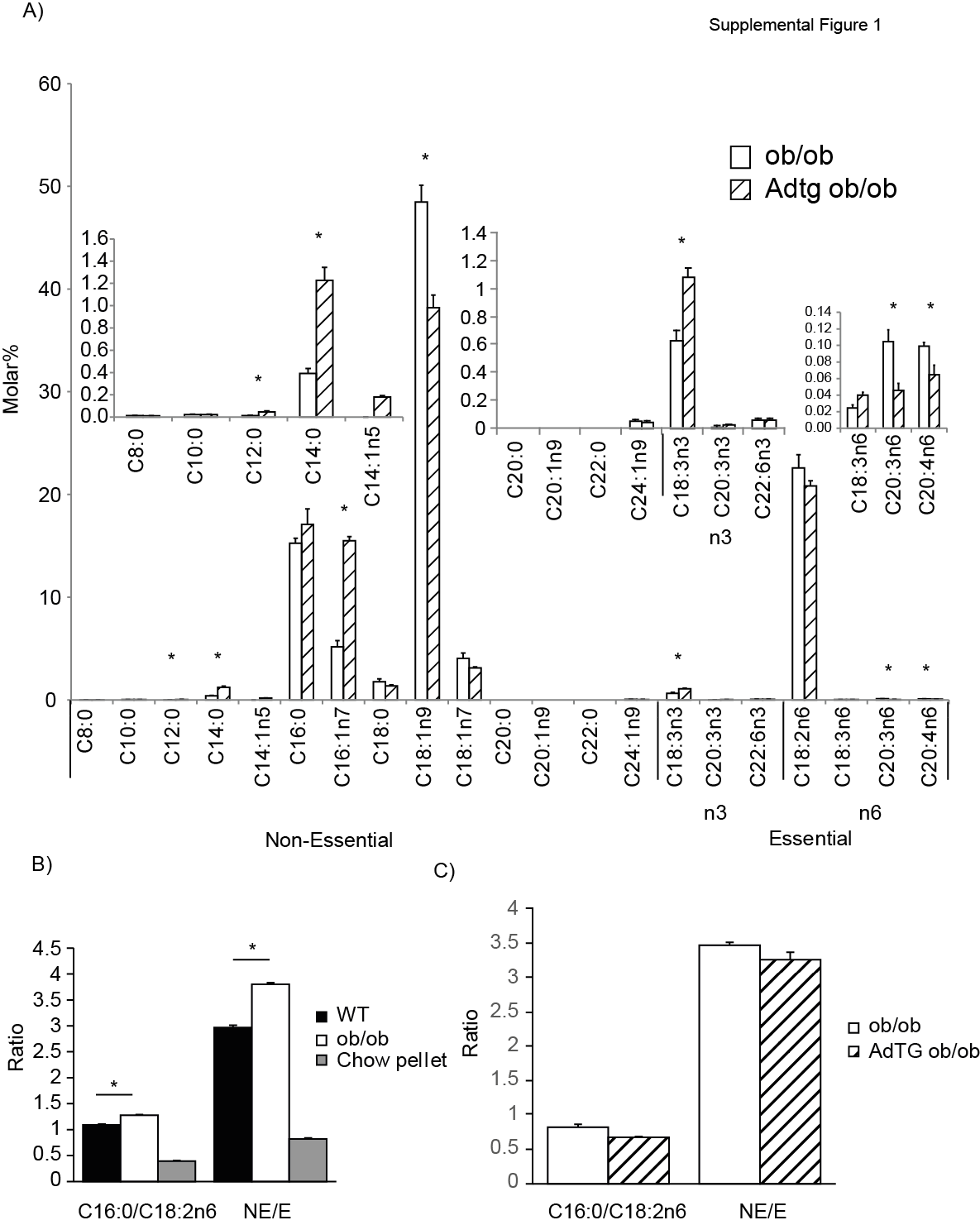


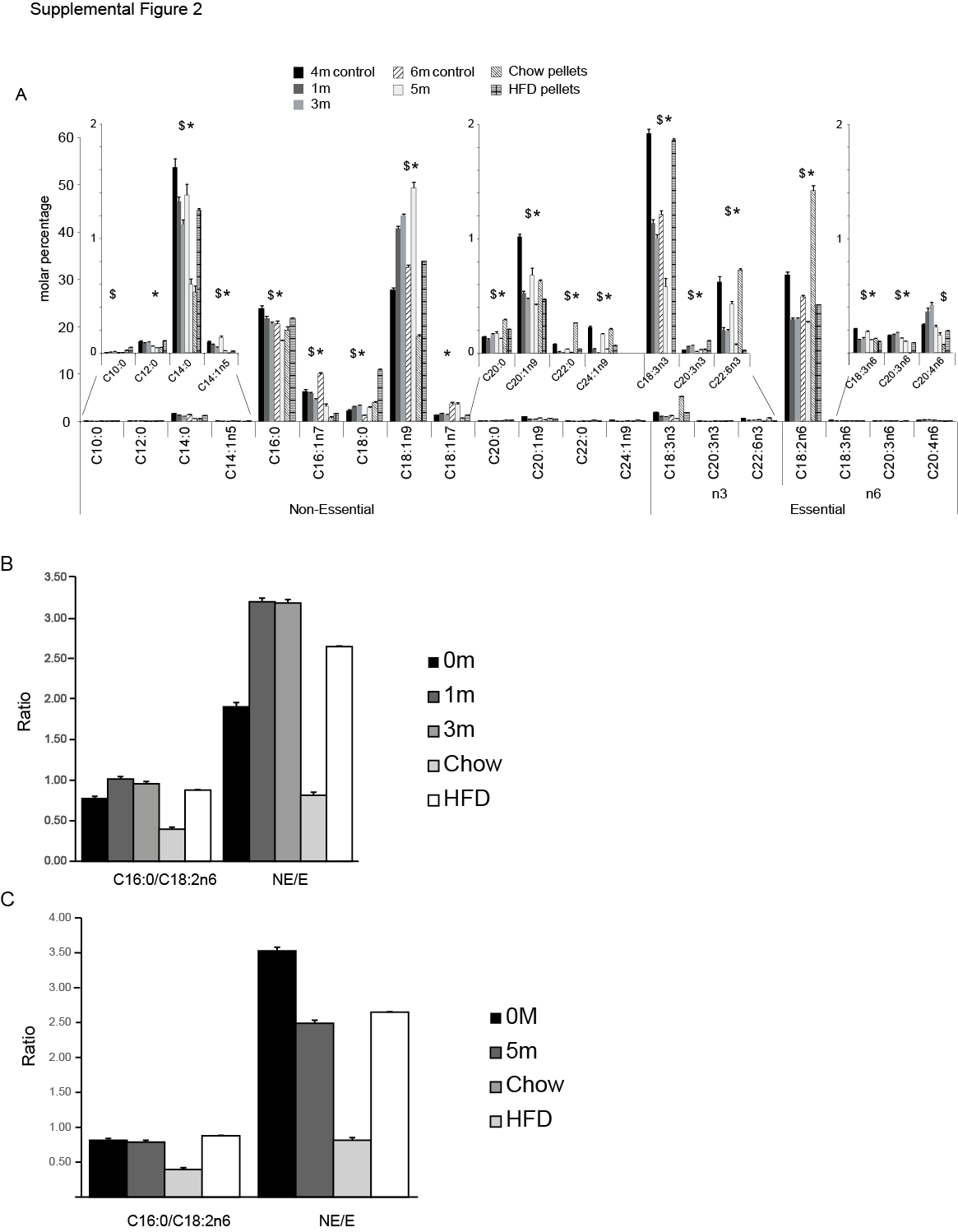


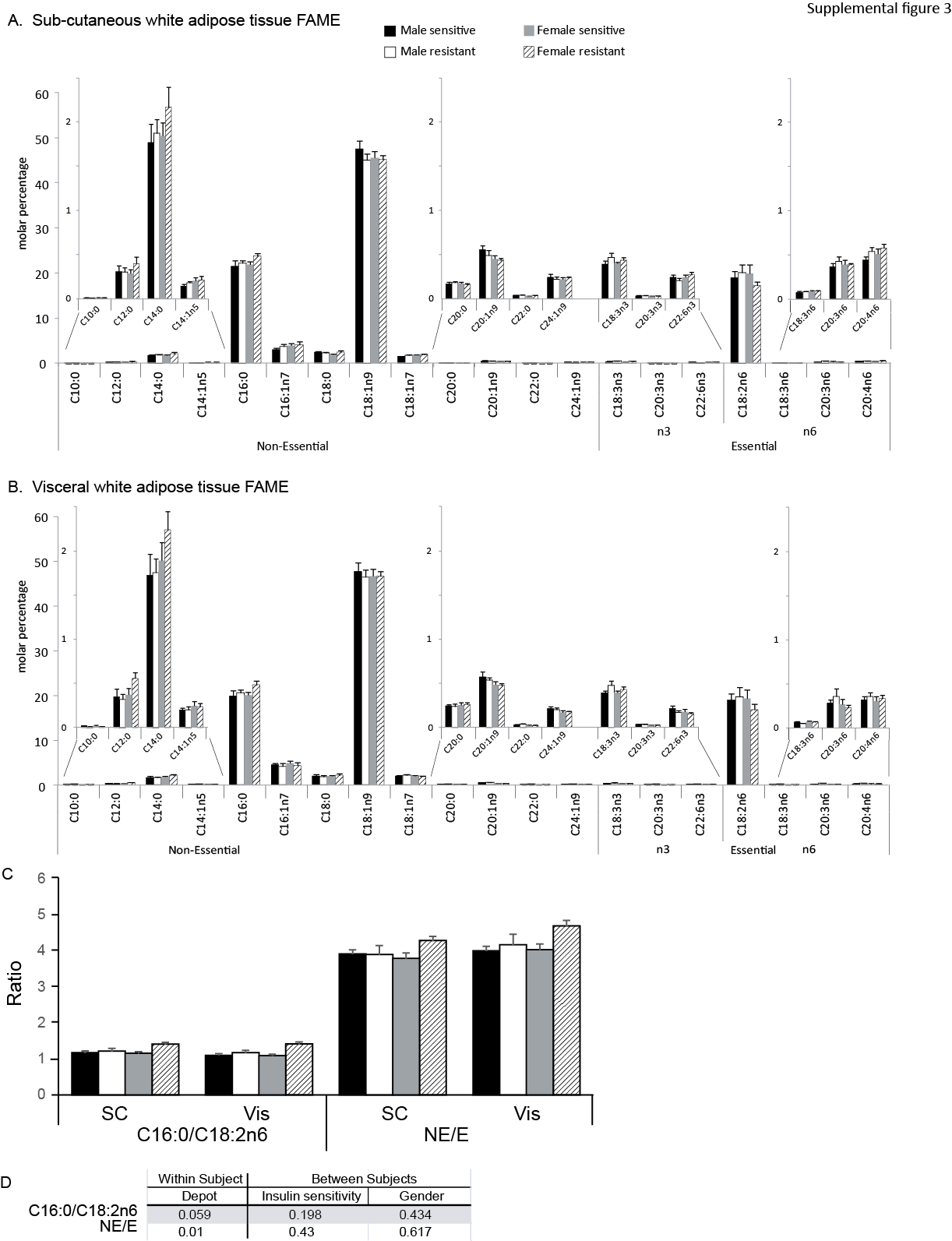

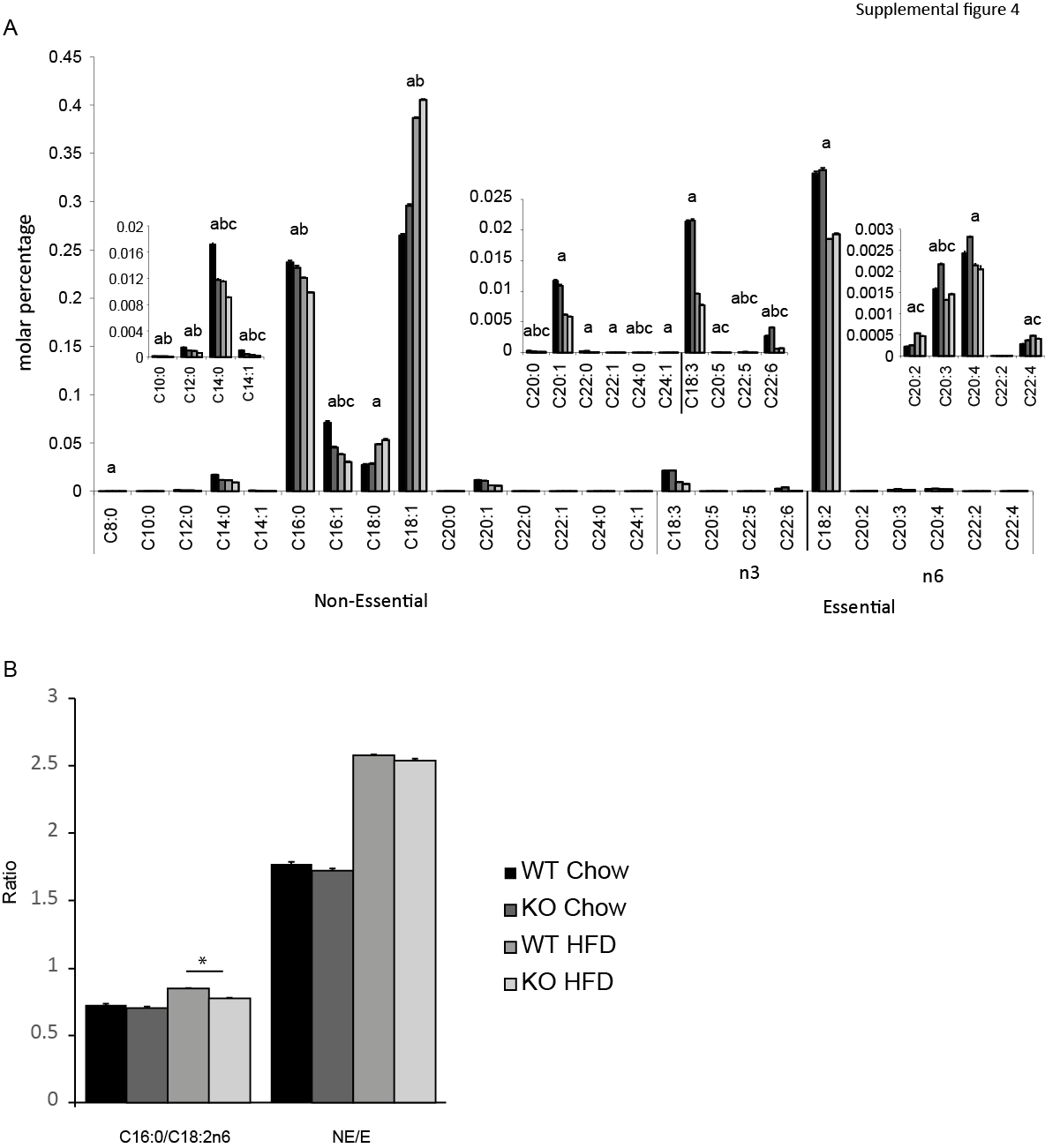


**
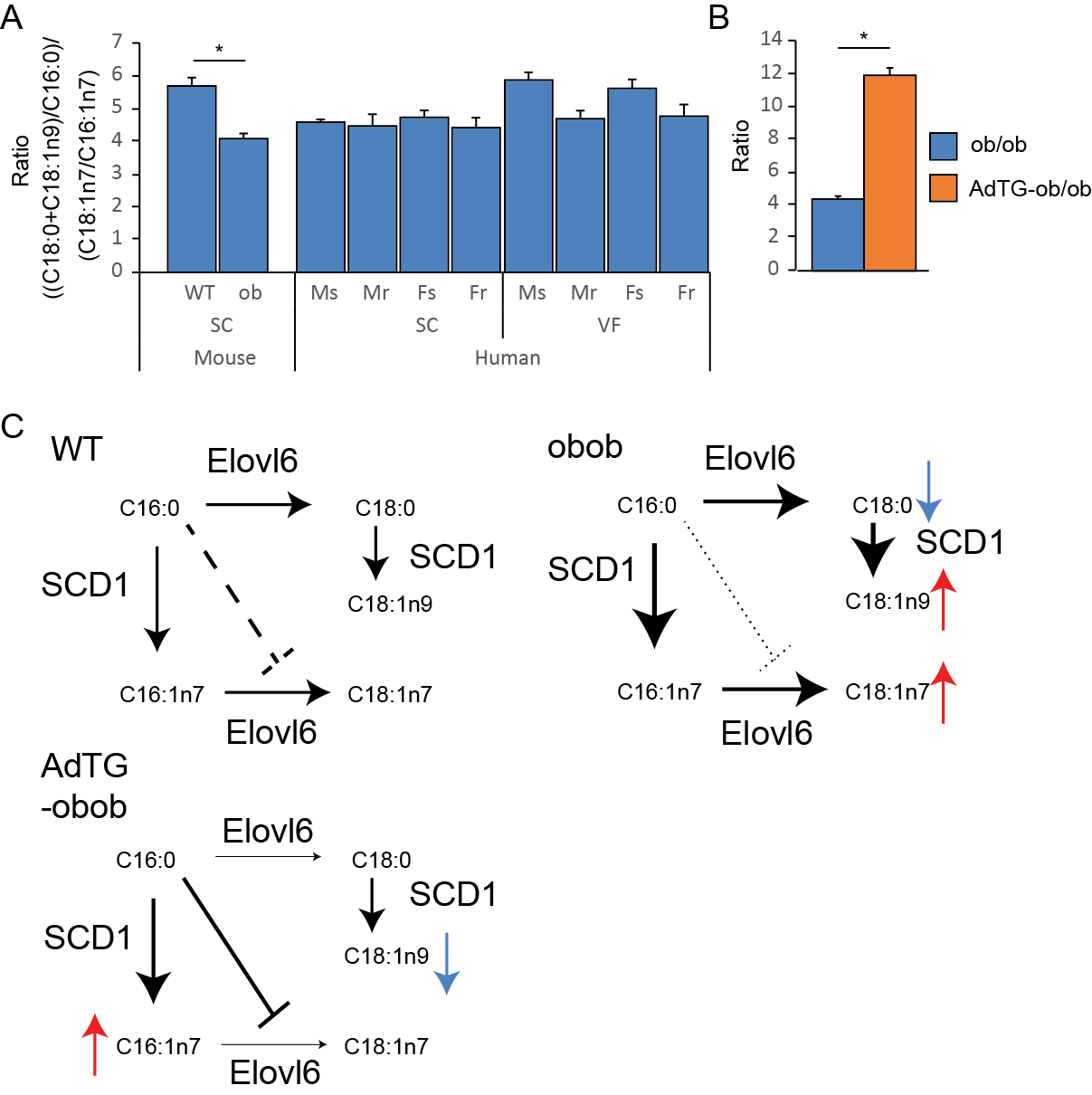
**

**Supplemental Figure 5**
